# Mapping small molecule binding data to structural domains

**DOI:** 10.1186/1471-2105-13-S17-S11

**Published:** 2012-12-07

**Authors:** Felix A Kruger, Raghd Rostom, John P Overington

**Affiliations:** 1 European Bioinformatics Institute, Wellcome Trust Genome Campus, Hinxton, UK

## Abstract

**Background:**

Large-scale bioactivity/SAR Open Data has recently become available, and this has allowed new analyses and approaches to be developed to help address the productivity and translational gaps of current drug discovery. One of the current limitations of these data is the relative sparsity of reported interactions per protein target, and complexities in establishing clear relationships between bioactivity and targets using bioinformatics tools. We detail in this paper the indexing of targets by the structural domains that bind (or are likely to bind) the ligand within a full-length protein. Specifically, we present a simple heuristic to map small molecule binding to Pfam domains. This profiling can be applied to all proteins within a genome to give some indications of the potential pharmacological modulation and regulation of all proteins.

**Results:**

In this implementation of our heuristic, ligand binding to protein targets from the ChEMBL database was mapped to structural domains as defined by profiles contained within the Pfam-A database. Our mapping suggests that the majority of assay targets within the current version of the ChEMBL database bind ligands through a small number of highly prevalent domains, and conversely the majority of Pfam domains sampled by our data play no currently established role in ligand binding. Validation studies, carried out firstly against Uniprot entries with expert binding-site annotation and secondly against entries in the wwPDB repository of crystallographic protein structures, demonstrate that our simple heuristic maps ligand binding to the correct domain in about 90 percent of all assessed cases. Using the mappings obtained with our heuristic, we have assembled ligand sets associated with each Pfam domain.

**Conclusions:**

Small molecule binding has been mapped to Pfam-A domains of protein targets in the ChEMBL bioactivity database. The result of this mapping is an enriched annotation of small molecule bioactivity data and a grouping of activity classes following the Pfam-A specifications of protein domains. This is valuable for data-focused approaches in drug discovery, for example when extrapolating potential targets of a small molecule with known activity against one or few targets, or in the assessment of a potential target for drug discovery or screening studies.

## Background

Research in the field of drug discovery is increasingly driven by the data mining of large-scale pharmacological, screening, patent, literature and other bioactivity data. Such approaches have led to interesting concepts that challenge historical dogma - for example the view that many small molecules and indeed drugs exert their effect through interactions with multiple rather than a single target [1]. New targets have been predicted for FDA approved drugs through analysis of large-scale bioactivity databases [2] and side-effect data mined from package inserts [3].

The discipline of combining small molecule bioactivity, the 'ligand space', with bioinformatics analyses of the 'target space' is also known under the name chemogenomics [4,5]. Chemogenomic approaches can be used to systematically examine and explore the binding of small molecules to large target families such as kinases [6,7] or G-protein coupled receptors (GPCRs) [8,9] or for the design of compounds targeting multiple proteins [10]. One of the current limitations of these approaches is the biased distribution of data that is available for individual targets. While there are a few prominent target classes such as certain GPCR families, protein kinases and various protease families, for which the bioactivity of many thousands of ligands has been measured, most targets have measured bioactivities for only a few compounds or no annotation at all [11]. To partially address this limitation, we propose an indexing of target space at a structural domain level, allowing aggregating ligands known to bind targets containing a given structural domain into a larger bioactivity class. The practical implication for the analysis of large-scale bioactivity data is a necessity to automatically and reliably annotate large numbers of protein targets with a domain containing the site of small molecule binding. We therefore propose to map small molecule binding to structural domains and present an initial implementation for targets in the ChEMBL database [12] (version chembl_13). Previous studies have statistically associated small molecule binding to protein domains [13] and direct mapping has been applied to ligands in crystallographic structures [14]. Here we extrapolate these mappings to pharmacologically relevant interactions described in the CHEMBL database.

Structural domains are independent folding units that form the basic evolutionary and architectural 'building blocks' of proteins [15]. While there can be large sequence differences between members of a domain family, the fold of the peptide backbone is generally conserved [16], even though (exceptional) cases of homologous proteins with differing folds have been identified and discussed [17]. A small protein would typically consist of one domain, while longer proteins are often an assembly of more than one domain [18]. In some eukaryotic proteins, the underlying intron-exon structure of the gene reflects this structural domain segmentation [19]. For the mapping of small molecule binding, targets consisting of combinations of domains impose a challenge because the binding site for the ligand might lie in either domain and in addition more than one domain in a protein might interact with the same or different ligands. Domain assignment information is available from a number of publicly available resources. SCOP [20] and CATH [21] are databases that define protein architecture based on hierarchical definitions of 3D structural domains. Pfam-A [22] is a database of hidden Markov chain models of non-overlapping full domain sequence alignments. Pfam-A domain definitions are also manually annotated and curated. Interpro [23] is a database that integrates different domain models into a comprehensive set of protein domains. For our purposes, the Pfam-A database with its non-overlapping, non-hierarchical architecture and extensive coverage of protein families, is ideal to map ligand binding to a given protein domain. In this study, we propose a simple heuristic to map the site of small molecule binding to Pfam-A domains and compare our results with binding site information from the protein sequence database Uniprot [24] and PDBe [25], a repository of crystallographic protein structures.

## Results

### Domain content of the human proteome and ChEMBL targets

**Figure 1:**
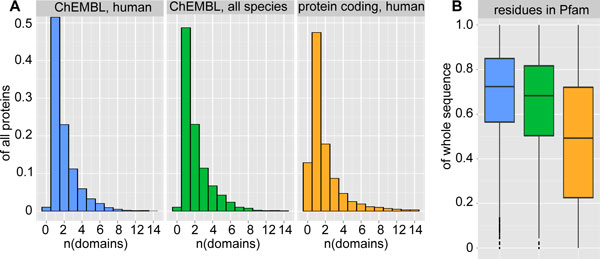
**Pfam domain content of drug targets**. (a) Shows the proportion of proteins having zero, one or more domains for all human targets in the ChEMBL target dictionary (blue), protein targets of all species in the ChEMBL target dictionary (green) and all protein coding genes (orange). (b) Barplots summarizing observed ratios of residues within a Pfam domain over the number of residues in the entire protein sequence for all human targets in the ChEMBL target dictionary (blue), protein targets of all species in the ChEMBL target dictionary (green) and all protein coding genes (orange). The median ratio for all protein coding genes is 0.50 and significantly lower than the corresponding ratio for targets in the ChEMBL data base (p < 2.2*10^-16^, Bonferroni adjusted for multiple testing).

The domain content of a human protein-protein interaction dataset has been described in a study by Patil *et al*. [26]. According to this work, 51 percent of all proteins from the interaction data set were found to contain more than one Pfam domain. We analyzed the Pfam domain content of the ChEMBL target dictionary and as a subset all human proteins within the ChEMBL target dictionary. We also queried the Ensembl database [27] (version: Ensembl65) for all protein coding genes in the human genome and analyzed the Pfam domain content for this set. The queries used to obtain this data are described in the Methods Section Code and queries. The results of this analysis are summarized in Figure 1. Additional file 1 provides a table with domain annotations for all targets in the analysis. Similar to Patil's interaction data set, 50.6 percent of the human targets in the ChEMBL target dictionary have more than one Pfam domain. In contrast, only 40.8 percent in the set of protein coding sequences from the human genome have more than one domain and 12.6 percent have no Pfam domain assigned. It appears therefore that while Patil's interaction set and the ChEMBL target dictionary are well covered by Pfam domain models, coverage of the entire set of human proteins is not complete.

In order to assess the impact of incomplete annotation for our set of ChEMBL targets, we determined for each target the number of residues belonging to a Pfam domain as a fraction of the number of residues in the overall protein sequence. We found that for the entire set of human proteins, the median of this fraction is 0.50 and about a quarter of all proteins have less than 20 percent of all residues assigned to a Pfam domain. The low ratio of residues within Pfam domains is likely due to incomplete coverage of Pfam-A models for the human proteome. For human protein targets in the ChEMBL database, the ratio of residues within Pfam domains is significantly higher (p < 2.2*10^-16^, Bonferroni adjusted for multiple testing): the median proportion of Pfam residues relative to sequence length is 0.72. In comparison, this ratio is 0.69 for all protein targets in the ChEMBL database, including non-human protein targets. Previous works suggests that proteins consist mainly of highly structured regions [20,21]. Therefore, we propose that coverage of Pfam-A domain annotation is almost complete for most ChEMBL targets but not for the entire set of human proteins. This is most likely due to the preference of drug discovery programs for well-characterized targets and the priority of disease-related proteins in functional and structural studies.

### Binding of small molecules within domain boundaries

Our attempt at mapping of ligand binding to discrete Pfam domains is based on the assumption that small molecule binding takes place within the structurally conserved region of a protein domain rather than in the surrounding non-Pfam domain regions. Following this premise, and assuming that the annotation with Pfam domains for our set of ChEMBL targets is complete, the mapping of small molecule binding is immediately achieved for proteins with a single domain. Thus, with our initial assumption, the heuristic covers 50% of all protein targets in the ChEMBL target dictionary. To estimate the accuracy of the outlined assumptions, we carried out systematic queries against UniprotKB/Swiss-Prot and PDBe and systematically evaluated the overlap of binding sites annotations and Pfam domain predictions. The Methods Section Code and queries describes the queries in detail.

**Figure 2:**
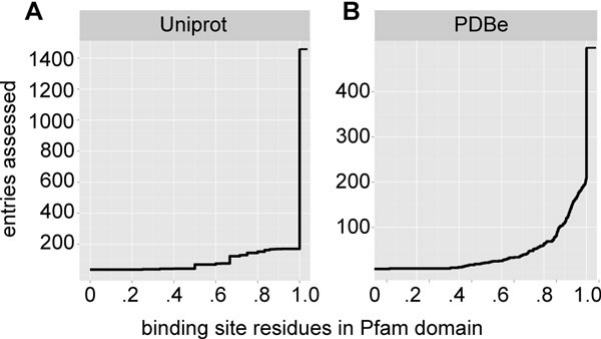
**Small molecule binding within Pfam domains**. (a) Shows how small molecule binding sites specified in Uniprot overlap with Pfam domains. The empirical cumulative distribution function describes the number of proteins for which the ratio of binding site residues within a Pfam domain over binding site residues outside of a Pfam domain is equal or greater to the value specified on the x-Axis. In analogy to the above (b) shows how small molecule binding sites specified in PDB motif overlap with Pfam domains.

UniprotKB/Swiss-Prot is a resource providing protein sequence and reviewed, manual annotation data. Binding site information is provided in the form of residue positions, in many cases focusing only on the most important residue(s). We queried Uniprot to retrieve all binding site information available for human protein targets in the ChEMBL database. The query was limited to human proteins to avoid a bias for targets with orthologs in the ChEMBL target dictionary, yielding binding site information for 1,428 targets. A comparison of binding site residue positions and Pfam domain boundaries revealed that 1,290 (88.4%) of annotated binding sites from UniprotKB/Swiss-Prot lie completely within a Pfam domain and only 36 (2.5%) entirely outside. Binding sites defined by a set of residues of which some are within and others outside of a Pfam domain are likely associated with the Pfam domain and therefore support our proposal that small molecule binding is associated to conserved sequence defined domains. The empirical cumulative distribution function (CDF) shown in Figure 2A describes the results of our query in greater detail. In analogy to the above, we queried the crystallographic structure data repository PDBe for binding site information and evaluated the overlap with Pfam domain predictions. Unlike in the manually annotated UniprotKB/Swiss-Prot, binding site information in PDBe is derived from molecular coordinates and encompasses all residues involved in a small molecule binding interaction. We retrieved binding site information for 496 human ChEMBL targets. Of all targets evaluated, 288 (58.1%) have all their binding site residues within a Pfam domain, and only 8 (1.6%) have all binding site residues outside any Pfam domain. The corresponding CDF is shown in Figure 2B. Compared to the CDF that represents the Uniprot query, there is a higher fraction of proteins having binding site residues both within and outside of Pfam domain boundaries. We attribute this to the greater detail of binding site annotation in PDBe, which encompasses all, rather than only one or few of the residues involved in ligand binding. Nevertheless, the majority of binding sites described in this analysis have a substantial number of residues within a Pfam domain, supporting our assumption that small molecule binding is associated to the defined and annotated regions in a protein that are detectable using Pfam domain models.

### Predicting binding sites for multi-domain proteins

Given that about half of all proteins in the ChEMBL target dictionary have more than one domain, we investigated ways to expand our mapping of small molecule binding from targets with only a single domain to targets with multiple domains. We had observed with high probability that small molecule binding in single domain protein takes place between the boundaries of a domain. We prepared a set of single domain protein targets from the ChEMBL data base by selecting each protein that had at least one ligand tested against it in a binding assay with a reported activity value less or equal 50 μM (see also Methods sections Mapping and Manual curation of input data). The occurrence of a domain in this set is thus a validation of a domain's potential to mediate a small molecule binding interaction. In the following, we consider all domains from this set as 'seed' domains with the potential to mediate small molecule binding. If such a 'seed' domain co-occurs with one or more 'non-seed' domains, our mapping defaults to this previously established seed domain. Hence, the mapping follows a heuristic based on the assumption that domains with known ligands take precedence over domains that do not occur in single domain proteins with known ligands. For example, in protein kinase Akt-3 (Q9Y243), which also contains a Pkinase_C and PH domain, the target of small molecule binding is the Pkinase domain. In total, our mapping covers 197,642 activities. A table with all mappings is provided in Additional file 2.

We benchmarked our mapping against manually curated binding site annotations from Uniprot and also against annotation extracted from crystallographic structures in PDBe. We queried the PDBe for protein structures with ligands matching our predictions and identified 217 entries that could be used to evaluate our mapping for multi-domain proteins. The comparison with binding site annotation retrieved from Uniprot was carried out in the same fashion and we identified 511 entries that could be used to evaluate the mapping. We considered predictions correct if at least half of all binding residues are located within the predicted domain. Details of the benchmarking are discussed in the Methods Section Validation. Unsurprisingly, the accuracy of predictions made for single domain targets is high (approx. 97 percent for both, the PDBe and Uniprot benchmark). For multi-domain proteins, the accuracy is at around 88 percent for both benchmarks. Benchmarking results of this validation are summarized in Table 1.

**Table 1:** Validation results

	single domain	multi domain
% correct Uniprot (N = 511)	97.53	87.22
% correct PDBe (N = 217)	97.64	88.88
# total predictions	1161	579

Benchmarking results against binding site information from Uniprot and PDBeMotif are summarized in the first and second row respectively. (N) specified in parentheses indicates how many targets were assessed in each benchmark. The total number of predictions made for single- and multi-domain targets is provided in the bottom row # total predictions.

**Figure 3:**
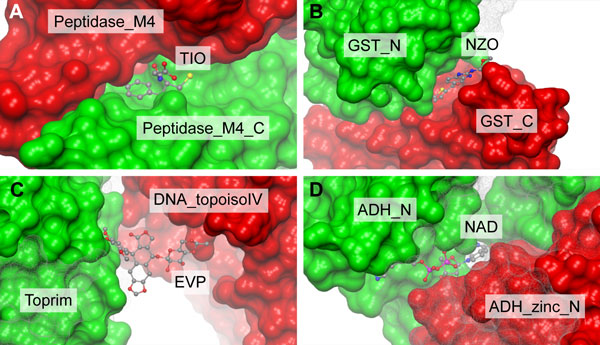
**Examples of small molecule binding at the interface of Pfam-A domains**. (a) Binding of thiorphan at the active site of thermolysin. The phenyl ring binds within the S1' pocket, the sulfur atom is coordinated with the active site Zinc atom (not shown). Thiorphan binds residues of both, the thermolysin metalloprotease catalytic domain (Peptidase_M4, red) and C-terminal domain (Peptidase_M4_C, green). (b) Nocodazole binding to the prostaglandin/GSH site of the human haematopoetic prostaglandin synthase D2. Nocodazole binds at the interface between the glutathione S-transferase N-terminal (GST_N, green) and C-terminal (GST_C, red) domains. (c) Etoposide binding to the DNA cleavage site of human type II DNA topoisomerase. The ligand binds residues both of the Toprim domain (green) and DNA topoisomerase IV domain (DNA_topoisoIV, red). (d) Nicotinamide-adenine dinucleotide binding to the active site of human alcohol dehydrogenase beta-1-beta-1 isoform. Binding takes place at the interface of the Alcohol dehydrogenase GroES-like domain (ADH_N, green) and Zinc-binding dehydrogenase domain (ADH_ZINC_N, red). Examples were rendered using PDB files 1zdp, 3ee2, 3qx3, 1u3u, respectively.

One limitation to our approach is its blindness towards ligand-binding Pfam domains that always occur in combination with at least one other Pfam domain. To account for the most important cases, we identified all Pfam domains that occur only in combination with other domains and occur one hundred or more times in the ChEMBL target dictionary. Based on a PDBe database survey of those domains (see Table 2), we selected the Pkinase_Tyr domain to be included in the list of seed domains. Another blind spot of this heuristic are ligand interactions that take place at the interface of two or more domains and thus are mediated by a combination of Pfam domains. In order to obtain an estimate of the importance of this type of interaction, we carried out a systematic query against PDBe to identify interactions that are reported in ChEMBL and fall into this category. We identified 12 targets (see Table 3), all of which are enzymes. Figure 3 shows four examples of small molecule binding at the interface of two domains. Additional files 3 and 4 contain graphics and session files for all 12 examples.

**Table 2:** Combinations of co-occurring validated domains

Domain	# ChEMBL targets	# PDB accessions
Neur_chan_memb	168	-
Pkinase_Tyr	159	148
fn3	126	-
Hemopexin	105	-
Ank	99	-

Listed are all domains that occur in multi-domain targets of the ChEMBL target dictionary at least one hundred times but never in a single domain target. The occurrence of each domain in the target dictionary is specified in the column # ChEMBL targets. The number of PDBe entries (if any) that describe small molecule interactions with any listed domain is indicated in the column # PDBe accessions.

**Table 3:** Small molecule binding at the interface of two or more Pfam-A domains

Domain combination	PDBe	ratio	# ChEMBL targets
ADH_N, ADH_zinc_N	1u3u [...]	0.58, 0.33	21
DNA_topoisoIV, Toprim	3qx3	0.32, 0.68	8
GST_C, GST_N	3ee2 [...]	0.62, 0.31	23
Hexokinase_1, Hexokinase_2	3goi [...]	0.56, 0.39	14
Mur_ligase_C, Mur_ligase	2am1 [...]	0.36, 0.36	6
NMT, NMT_C	1iyk [...]	0.46, 0.41	6
OTCace, OTCace_N	1oth	0.50, 0.50	3
Peptidase_M4, Peptidase_M4_C	1zdp [...]	0.50, 0.50	2
Peptidase_S9, DPPIV_N	3d4l [...]	0.51, 0.43	10
Topoisom_I, Topo_C_assoc, Topoisom_I_N	1k4t [...]	0.35, 0.31, 0.35	5
S-AdoMet_synt_N, S-AdoMet_synt_C	1o93	0.41, 0.43	2
Tubulin, Tubulin_C	1ia0	0.30, 0.48	20

Summary of small molecule binding mediated through more than one domain. Combinations of Pfam-A domains are specified in the left-most column. The column PDBe provides one exemplary structure accession ([...] indicates that more entries exist) and 'ratios' specifies the ratio of binding site residues within the corresponding domain over all binding site residues. # ChEMBL targets indicates the number of targets in ChEMBL containing a given domain combination.

### Small molecule binding to Pfam domains from a chemogenomic perspective

**Figure 4:**
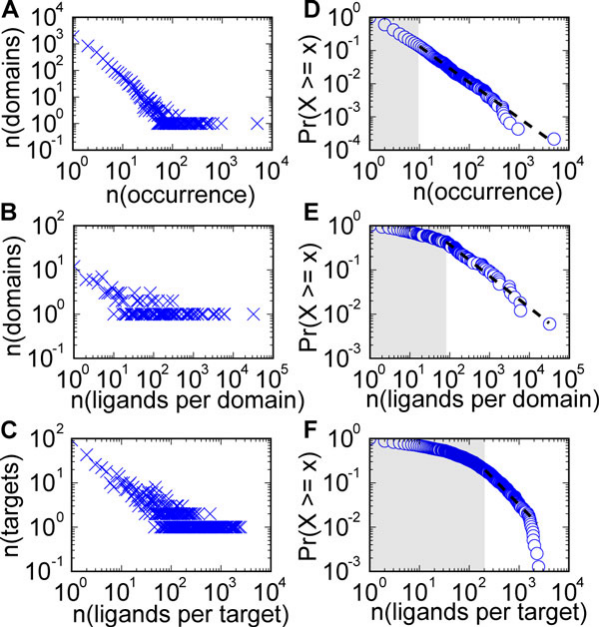
**Power-law distribution of Pfam domains and small molecule ligands**. (a-c) Log-log plots of observed distributions. X-Axis: proteome frequency of Pfam-A domains (a), ligands per Pfam domain (b), ligands per target (c). y-Axis: Number of instances with count X equal to corresponding x-value. (d-f) Corresponding cumulative distribution functions overlaid with the fit of the power-law distribution (dotted line). X-Axis: proteome frequency of Pfam-A domains (d), ligands per Pfam domain (e), ligands per target (f). Y-Axis: Proportion of instances with count X equal to or greater than x.

We used the mapping described in the previous sections to analyze the numbers of ligands tested for individual Pfam families and set these in a relationship with the genomic frequencies of Pfam domains. Table 4 lists the numbers of ligands tested in binding assays against the top 10 Pfam domains. These 10 domains cover almost three quarters of all reported binding events (63,070 of 84,891). This highly skewed distribution is reminiscent of the genomic frequency distributions of Pfam domains. The frequency of protein domains in bacterial and eukaryotic genomes follows a power-law distribution [28,29] with a small number of very frequent domains while the vast majority of domains has only few occurrences. In this study, we examined the distribution of occurrences of Pfam domains in human protein coding genes as well as the distribution of known ligands per Pfam domain following a state-of-the-art protocol [30]. According to this protocol, we calculated the scale-parameter alpha and the smallest number of occurrence (xmin) to which the power-law still applies. We then used Kolmogorov-Smirnov testing (KS) to estimate the goodness-of-fit. The resulting p-Value is a measure for the plausibility of a power-law hypothesis. In a final step, we used a maximum likelihood ratio test to compare the power-law with alternative hypotheses. Figure 4 and Table 5 summarize the results of our analysis, which is described in the Methods section Statistical analysis of power-law distributions. The results of our analysis confirm that the distribution of Pfam domains in the set of protein coding genes follows a power-law. A power-law equally applies to large parts of the distribution of known ligands per Pfam domain, covering a range of observed instances (ligands per domain family) that is shifted up one order of magnitude compared to Pfam domain frequencies. In contrast to the distribution of Pfam domains and known ligands per Pfam domain, the highest numbers of ligands for individual proteins appear to be limited and the number of targets with very few ligands is smaller than would be expected by a power-law. The frequency of Pfam domains in the human genome is dictated by gene duplication under selective pressure and models describing this process have been presented previously [31,32]. In analogy, the distribution of known small molecule ligands for Pfam domains is shaped by the slow and incremental exploration of target classes in drug discovery. Target families with known ligands are more likely to gain new ligands, for example through lead-optimization studies and selectivity screens. Once a target is economically exploited, lead-optimization projects are halted (hence the apparent upper limit for individual targets) or directed towards other targets within the same family. Target families without known ligands on the other hand only become subject to investigation if extensive scientific evidence suggest favorable future outcomes.

**Table 4:** Pfam domains with most ligands tested in binding assays

Pfam-A	# cmpds
7tm_1	32060
Pkinase	5989
Pkinase_Tyr	5858
Hormone_recep	4239
SNF	3399
Trypsin	3172
Ion_trans	3107
Peptidase_C1	1760
Asp	1757
adh_short	1729

Shown are the 15 Pfam-A domains with most associated ligands. Pfam-A specifies the domain name, #cmpds the number of ligands tested in binding assays with potency no weaker than 50 μM.

**Table 5:** Statistical analysis of power-law parameters

	Frequency of Pfam domains	Ligands per Pfam domain family	Ligands per target
xmin	10	81	210
alpha	2.07	1.71	2.15
Goodness of fit	0.5	0.42	0.42
vs_lognormal	yes (p = 5.1*10^-9)	yes/no (p = 0.48)	yes/no (p = 0.57)
vs_exponential	yes (p = 3.9*10^-3)	yes (p = 0.10)	yes (p = 8.5 *10^-8)
vs_weibull	yes (p = 2.1*10^-4)	Yes/no (p = 0.16)	no (p = 1.0*10^-3)
magnitude	~ 3	~ 3	~ 1
support for power-law	yes	yes	no

Parameters of the power-law functions fitted to the observed distributions of Pfam-A domain frequencies (left column), number of ligands associated with each Pfam-A domain (middle column) and number of ligands associated with individual targets (right column) are shown in columns 'xmin' and 'alpha'. 'Goodness of fit' indicates the p-Value calculated from a KS goodness of fit test. The rows vs_lognormal, vs_exponential, vs_weibull indicate outcomes of maximum-likelihood tests against alternative distributions. 'Yes' indicates significant support for a power-law distribution, 'no' indicates support for the alternative over a power-law. 'Magnitude' specifies the orders of magnitude in the distribution spanned by a power-law and 'support for power-law' is the summary outcome for each distribution.

### Ligand sets

**Figure 5:**
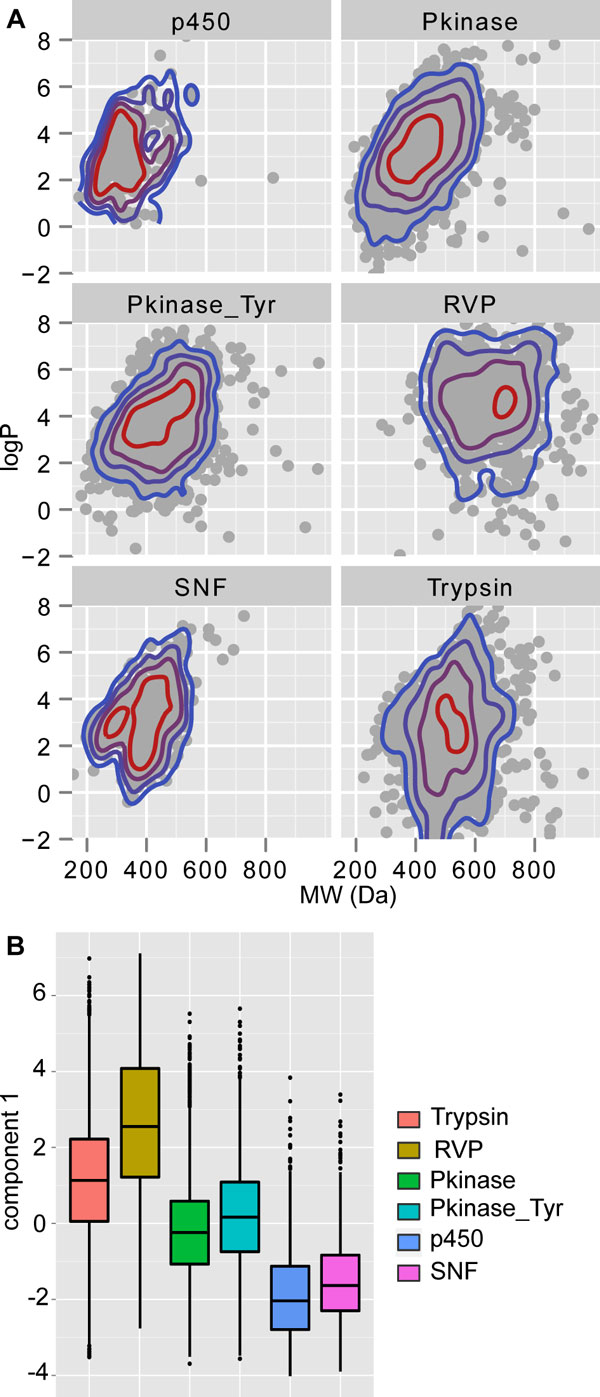
**Chemical space of the ligands of 6 target classes**. (a) Plotted are molecular weight vs logP for ligands of 6 target classes. Colored rings outline the ligand densities at any given point in projected chemical space, where densities halve for each ring traversing the scale from red to blue. (b) Projection of the values of the first principal component calculated for ligands of the 6 selected target classes. Distributions are distinct for each target class (p = 2.2*10^-16 ^for all combinations, with the exception of the comparison between the p450 and SNF domain classes, where p = 1.2*10^-13^; all p-Values were calculated using Bonferroni correction for multiple testing). RVP - retroviral aspartyl protease; Pkinase - protein kinase; Pkinase_Tyr - tyrosine kinase; p450 cytochrome - p450; SNF - sodium neurotransmitter symporter, Trypsin - serine protease trypsin.

We conducted a survey of the chemical space occupied by ligands of given Pfam domains. Here, we focus on 6 Pfam domains of high relevance for drug discovery, protein kinase (Pkinase), tyrosine kinase (Pkinase_Tyr), cytochrome p450 (p450), retroviral aspartyl protease (RVP), sodium neurotransmitter symporter (SNF) and the serine protease trypsin (Trypsin). For each of these, Figure 5A depicts the chemical space of known ligands in terms of two simple descriptors, molecular weight and the calculated partitioning coefficient logP. Specifically, these plots show the relative density of ligands at a given point in projected chemical space. It is obvious from the overlap on these plots that true separation of ligands cannot be achieved based solely on these two descriptors. However, judging from the distinct distribution peaks for ligands of each domain it is conceivable that probability density functions for combinations of simple descriptors could enable target class prediction based on chemical structure. To explore this further, we used six basic molecular descriptors of all ligands associated with either of the chosen domains as input for a principal component analysis (PCA, see Methods section Principal Component Analysis and Additional file 5 for details). Those six descriptors are molecular weight, the oil/water partition coefficient (ALogP, calculated following the method of Ghose and Crippen [33]), polar surface area (PSA, following the method of Ertl [34]), the number of rotable bonds, the number of hydrogen bond donors and the number of hydrogen bond acceptors. In preparation for the PCA we removed from each set of descriptor values the distribution outliers and subsequently scaled all values to unit variance. The loadings of components obtained after PCA are summarized in Table 6. We used the first principal component to project the molecular variability of ligand sets onto one-dimensional distributions of component values for each domain family (see Figure 5B). We observed that the obtained distributions reflect the relationships between different domain families. For example, there is a relatively large overlap between ligand sets of the related Pkinase and Pkinase_Tyr domains, which are both dominated by analogues of the kinase substrate adenosine triphosphate. Pair-wise Student t-tests confirmed that the descriptor spaces of ligands for each domain class are distinct (p < 2.2*10^-16 ^for all combinations, with the exception of the comparison between the p450 and SNF domain classes, where p = 1.2*10^-13^; all p-Values were calculated using Bonferroni correction for multiple testing). Hence, the distributions obtained here can be exploited to assess the binding potential of a given small molecule to a Pfam domain family, based on a combination of simple descriptors. To retain predictive power, this approach requires that the scope of the search is limited to a selection of domains. Prior knowledge about a given small molecule can instruct the selection of domains, for example if information about co-localization with protein complexes is available.

**Table 6:** Loadings of the principal components

	PC1	PC2	PC3	PC4	PC5	PC6
logP	0.0065	-0.8068	0.2363	-0.3698	0.2969	-0.2614
Molweight	0.4670	-0.3361	-0.0544	-0.1498	-0.6339	0.4916
HBD	0.3965	0.2750	0.7574	-0.0600	0.3267	0.2979
HBA	0.4498	0.0944	-0.6080	-0.2818	0.5498	0.1931
RTB	0.4332	-0.2790	-0.0448	0.8250	0.1320	-0.1857
PSA	0.4844	0.2716	0.0370	-0.2780	-0.2889	-0.7276

Shown are the factor correlation coefficients for individual components.

## Conclusions

In this study, we show that small molecule binding sites are associated with the regions in a protein that map to a Pfam domain, and hence typically have a discrete structure defined by a conserved sequence profile. We exploit this knowledge to map small molecule binding to Pfam domains in single- and multi-domain proteins. The integration of small molecule bioactivity data from the ChEMBL database and (predicted) structural data from Pfam will drive cross-linking across databases and deeper semantic annotation for chemical biology. In addition, our mapping allowed for an analysis of the distribution of known small molecule ligands per Pfam domain. The power-law behavior of this distribution mirrors the genomic distribution of protein folds and the incremental progression of drug discovery.

The heuristic presented here is simple and efficient. However, the mapping does not address two naturally occurring edge cases. Firstly, a number of Pfam domains occur only in combination with other domains and hence are not picked up in the initial seeding step. We address this partially by manually including such domains if they occur in more than one hundred ChEMBL targets. The second case is the relatively rare occurrence of ligand binding at the interface of domains, as discussed in the section on mapping small molecule binding to multidomain proteins.

**Figure 6:**
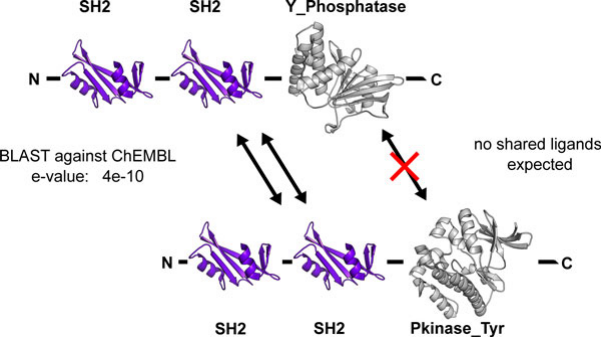
**Domain poisoning in chemogenomics queries**. (a) The schematic shows the domain structure of a protein in a hypothetical query - the rat Tyrosine-protein phosphatase Syp (P35235) - and one of the hits, retrieved from a BLAST query against the ChEMBL target dictionary - the rat Tyrosine-protein kinase SYK (Q64725). The relatively low expectation value for this query comes from high scoring alignments of the SH2 domains. At the same time, the overlap between small molecules binding both proteins is expected to be low.

One incentive to annotate recorded activities of small molecules against multi-domain proteins is a phenomenon we term 'domain poisoning' - where the presence of a common 'spectator domain' links together targets on the basis of sequence searches, but the ligand-binding domain is absent from the identified homologue. To avoid false positives, we were previously forced to use very conservative cut-offs for sequence similarity (see [35] for an example) because we found that without this safeguard, known drug targets were associated with a query protein through high conservation in regions that are not involved in small molecule binding and thus 'poisoned' our query results with irrelevant compounds. For example, when querying the ChEMBL target dictionary for targets similar to Tyrosine-protein phosphatase Syp (e.g. P35235), the presence of SH2 domains would result in relatively strong association with tyrosine kinases such as Tyrosine-protein kinase SYK (e.g., Q64725) and poison the query with kinase inhibitors (see Figure 6 for an illustration). In such a case, a query using only the domain relevant to small molecule binding would automatically filter out targets that are associated through domains not relevant to ligand binding.

The mapping described in this study further provides ligand sets for the development of methods to predict bioactivity for new compounds and gives an estimate of the chemical space of ligands associated with each domain. We also used these sets as a starting point to explore the selectivity of small molecules within and across protein families following the Pfam domain definitions. Mappings and ligand sets resulting from this study will be kept up-to-date with new ChEMBL releases and are available at http://www.ebi.ac.uk/~fkrueger/mapChEMBLPfam, along with documentation.

## Methods

### Mapping

Practically, the mapping was carried out as follows. For all targets in the ChEMBL target dictionary, we collected activities measured in binding assays that are linked directly and unambiguously to a single target. (Assay type = B, multi- and complex-flags = 0) The activity type was required to be either of the following: Ki, Kd, IC_50_, EC50, -Log Ki, pKd, pA2, pI, pKa. We further filtered out all activities weaker than 50 μM. The remaining mappings were kept and a dictionary of validated domains created. Multi-domain proteins were scanned for the presence of validated domains and categorized as either of the following. i) No validated domain, ii) only one validated domain (or multiple copies thereof), iii) more than one validated domain. Case i) results in no mapping, case ii) assigns all ligands to the validated domain. In the case iii) that more than one validated domain occurs in a protein we did not assign any mapping. A summary of all co-occurrences of validated Pfam-A domains is provided in Additional file 6.

### Validation

Validation was carried out against data from Uniprot as well as PDBe. Uniprot lists manually curated positions of residues that participate in ligand binding while information about residues in close proximity to the bound ligand can be extracted from PDBe using the algorithm PDBeMotif [36]. Binding site annotations from PDBeMotif contain explicit information about the ligand, in the form of a three-letter code, and the residue numbers of interacting residues in the target protein. We can thus assess binding within Pfam domain boundaries by comparing the position of each binding site residue with the start and end positions of a given domain. Predictions on multi-domain proteins were benchmarked by calculating the fraction of residues within a predicted domain over all residues involved in the binding of the corresponding ligand. The resulting ratio can be considered as a measure of association between a predicted Pfam domain and ligand binding, with high values indicating strong associations and vice versa. We argue that a value of 0.5 or greater is a robust measure of association between a Pfam-A domain and ligand binding. Accordingly, predictions benchmarked against Uniprot or PDBe were either classified as correct if this ratio was equal or greater than 0.5 or classified as false if this ratio was less than 0.5.

### Manual curation of input data

In some few cases, small molecule bioactivities reported in ChEMBL are mapped to Uniprot identifiers that represent fragments of a protein. This might be due to annotation errors, or the lack of a Uniprot entry representing the full-length protein. These cases can be problematic for our mapping. As an example, some activities extracted from an article on phosphodiesterase inhibitors (PubMed 8027992) map to the Uniprot identifier Q864F1. This identifier represents an N-terminal fragment of the pig phosphodiesterase 5, containing only the GAF domain and, crucially, missing the PDEase_I domain. Thus, small molecule binding is incorrectly mapped to the GAF domain. We identified five critical protein fragments in the ChEMBL target dictionary and removed these manually before applying our mapping algorithm. A list of these targets and justification for removal is provided in Additional file 7.

### Statistical analysis of power-law distributions

Statistical analysis was carried out in R [37] unless otherwise stated. The protocol we followed to test the distributions of Pfam domain occurrences and number of known ligands for a power-law behavior comprises 4 steps. In the first step, we use the R package plfit.R to determine the scale parameter α and xmin. We then use the package powerlaw.R http://www.rickwash.com/papers/cscw08-appendix/powerlaw.R to calculate the goodness-of-fit and corresponding p-Value. For the maximum-likelihood calculations we use the functions pareto.lnorm.llr, pareto.exp.llr and pareto.weibull.llr. Visualizations were created using the script plplot.py. All functions except powerlaw.R were provided by Aaron Clauset and Cosma Shalizi http://tuvalu.santafe.edu/~aaronc/powerlaws/.

### Principal component analysis

We selected ligands from mappings for 6 Pfam domains and retrieved pre-calculated descriptor values from the compound_properties table within the ChEMBL database. To prepare the data for scaling to unit variance, we excluded as outliers the first and hundredth percentile of each descriptor value distribution (see Additional file 5). Scaling to unit variance and principal component analysis was carried out using the R function prcomp.

### Code and queries

The workflow for this study was implemented in python and R. The code is deposited at https://github.com/fak/mapChEMBLPfam. Pfam domain annotations and estimated domain boundaries for all protein entries were retrieved from http://pfam.sanger.ac.uk/protein/X?output=xml where X is the Uniprot accession of a query protein. The corresponding function can be found as getPfamDomains.py in the code repository. Binding site annotations from Uniprot were retrieved from http://www.uniprot.org/uniprot/X.xml, where X is the Uniprot accession of a ChEMBL target. Residues in close proximity to the bound ligand were retrieved from PDBeMotif using a query submitted to http://www.ebi.ac.uk/pdbe-site/pdbemotif/hitlist.xml. The corresponding deposited functions are called queryUniprot.py and queryPDB.py, respectively. We used SIFTS [38] to translate between PDBe and Uniprot residue coordinates. Protein coding genes in the human genome were extracted from Ensembl using Ensembl Biomarts [39] with the deposited function queryBioMaRt.R.

## Competing interests

The authors declare that they have no competing interests.

## Authors' contributions

Conceived and designed the experiments: JPO, FAK. Implemented the mapping: FAK. Analyzed the data: FAK, RR. Wrote the paper: JPO, FAK.

## Supplementary Material

Additional file 1 **Domain annotations for the ChEMBL target dictionary**. Tab-delimited file. The column 'uniprot' provides the Uniprot identifier, the column 'pfam' the Pfam identifier, 'start' and 'end' the start and end positions of the respective domain in the Uniprot sequence. Click here for file

Additional file 2 **Table of mapped interactions**. Tab-delimited file. The column 'activity' provides the value of the ChEMBL field activity_id, 'domain' the mapped Pfam-A domain, 'molregno' the identifier for the small molecule, 'uniprot' provides the Uniprot identifier and 'maptype' indicates whether the protein target is a single- or multi-domain protein. Click here for file

Additional file 3 **Renderings of small molecule binding at the interface of Pfam-A domains**. This is a folder containing graphics in JPG format. Click here for file

Additional file 4 **Session files of small molecule binding at the interface of Pfam-A domains**. Zipped folder containing Qt-MG session files and required PDB files. Click here for file

Additional file 5 **Outlier selection for PCA**. Boxplots show distributions of descriptor values for all molecules in the analysis. Red lines indicate chosen cut-offs for outlier selection. Click here for file

Additional file 6 **List of conflicts between 'seed' domains occurring within the same target**. Tab delimited file. The column 'uniprot' provides the Uniprot identifier, the column 'conflict' the seed domains co-occurring in the specified protein. Click here for file

Additional file 7 **Manually removed protein targets**. This is a text file listing all entries that were manually removed before mapping small molecule binding. Reasons for the removal are indicated for each identifier. Click here for file
