# Supplementary figures and images for: Mapping small molecule binding data to structural domains
Source: BMC Bioinformatics. 2012 Dec 7;13(Suppl 17):S11. doi: 10.1186/1471-2105-13-S17-S11 (PMC3521243; doi:10.1186/1471-2105-13-S17-S11)

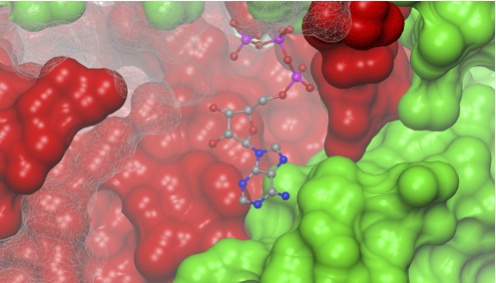

Supplement: Additional file 3 — Renderings of small molecule binding at the interface of Pfam-A domains. This is a folder containing graphics in JPG format. [file 1471-2105-13-S17-S11-S3.zip › additional_file_4/AdoMetClose.jpg]

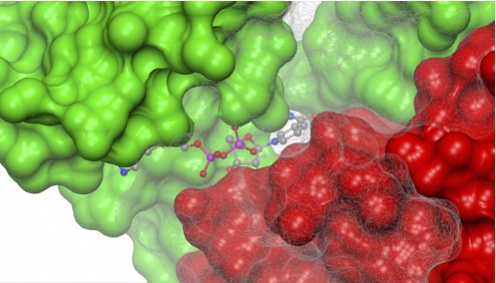

Supplement: Additional file 3 — Renderings of small molecule binding at the interface of Pfam-A domains. This is a folder containing graphics in JPG format. [file 1471-2105-13-S17-S11-S3.zip › additional_file_4/alcDehydrogenaseClose.jpg]

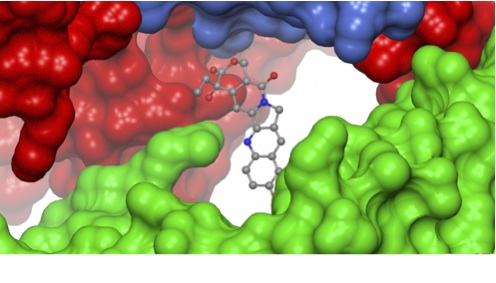

Supplement: Additional file 3 — Renderings of small molecule binding at the interface of Pfam-A domains. This is a folder containing graphics in JPG format. [file 1471-2105-13-S17-S11-S3.zip › additional_file_4/dnaTopoisomeraseIClose.jpg]

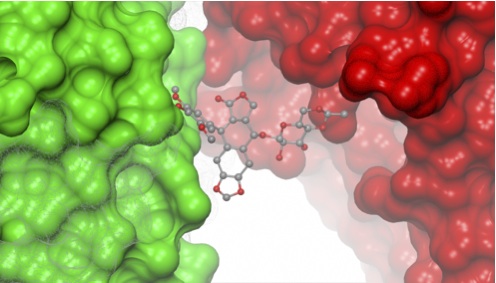

Supplement: Additional file 3 — Renderings of small molecule binding at the interface of Pfam-A domains. This is a folder containing graphics in JPG format. [file 1471-2105-13-S17-S11-S3.zip › additional_file_4/dnaToposomeraseClose.jpg]

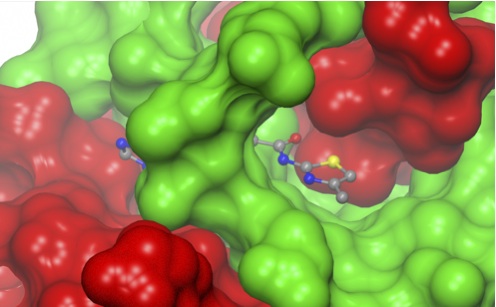

Supplement: Additional file 3 — Renderings of small molecule binding at the interface of Pfam-A domains. This is a folder containing graphics in JPG format. [file 1471-2105-13-S17-S11-S3.zip › additional_file_4/hexokinaseClose.jpg]

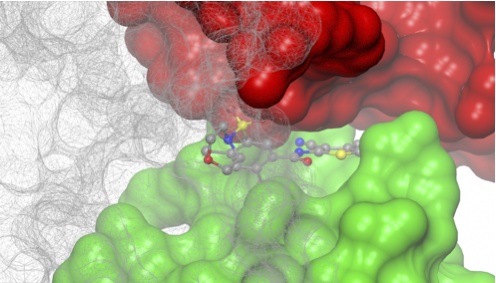

Supplement: Additional file 3 — Renderings of small molecule binding at the interface of Pfam-A domains. This is a folder containing graphics in JPG format. [file 1471-2105-13-S17-S11-S3.zip › additional_file_4/murLigaseClose.jpg]

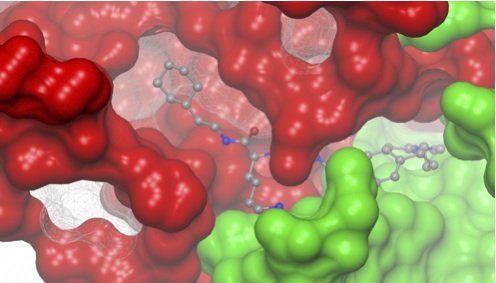

Supplement: Additional file 3 — Renderings of small molecule binding at the interface of Pfam-A domains. This is a folder containing graphics in JPG format. [file 1471-2105-13-S17-S11-S3.zip › additional_file_4/myrTransClose.jpg]

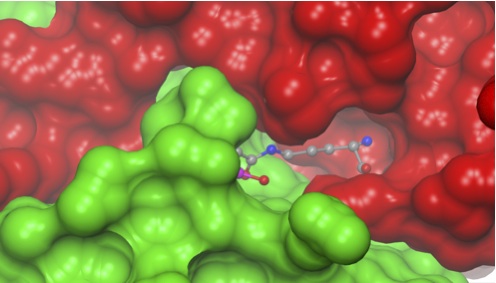

Supplement: Additional file 3 — Renderings of small molecule binding at the interface of Pfam-A domains. This is a folder containing graphics in JPG format. [file 1471-2105-13-S17-S11-S3.zip › additional_file_4/OTCaceClose.jpg]

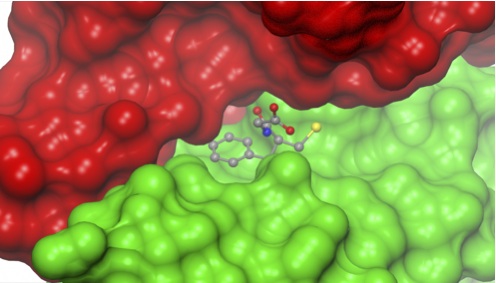

Supplement: Additional file 3 — Renderings of small molecule binding at the interface of Pfam-A domains. This is a folder containing graphics in JPG format. [file 1471-2105-13-S17-S11-S3.zip › additional_file_4/peptidaseM4Close.jpg]

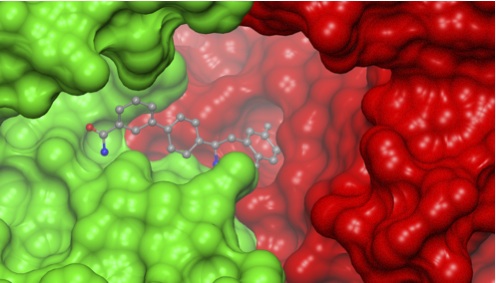

Supplement: Additional file 3 — Renderings of small molecule binding at the interface of Pfam-A domains. This is a folder containing graphics in JPG format. [file 1471-2105-13-S17-S11-S3.zip › additional_file_4/peptidaseS9Close.jpg]

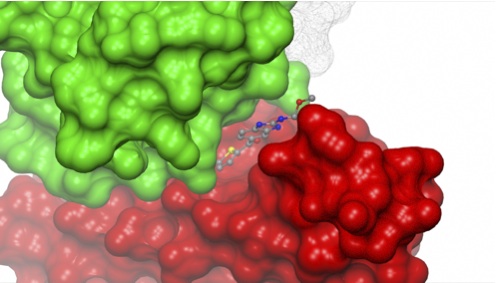

Supplement: Additional file 3 — Renderings of small molecule binding at the interface of Pfam-A domains. This is a folder containing graphics in JPG format. [file 1471-2105-13-S17-S11-S3.zip › additional_file_4/pglandinSynthaseClose.jpg]

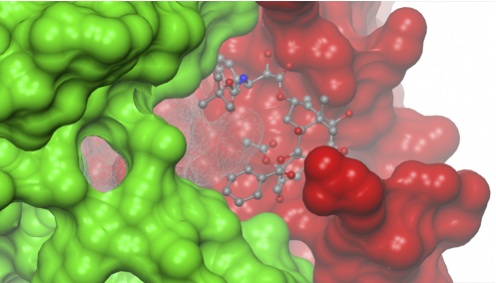

Supplement: Additional file 3 — Renderings of small molecule binding at the interface of Pfam-A domains. This is a folder containing graphics in JPG format. [file 1471-2105-13-S17-S11-S3.zip › additional_file_4/tubulinClose.jpg]

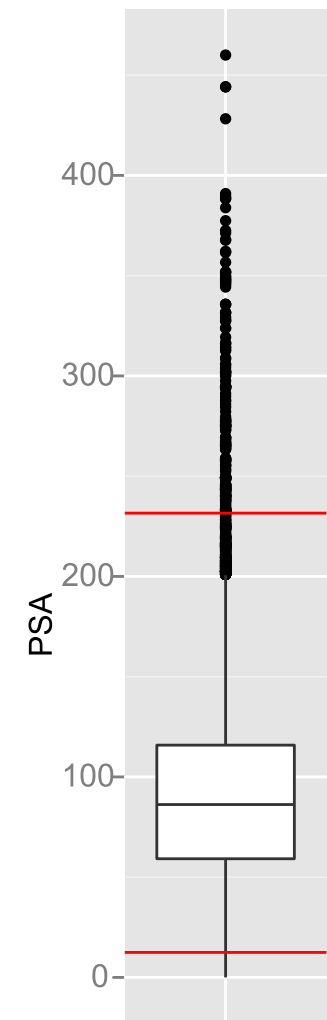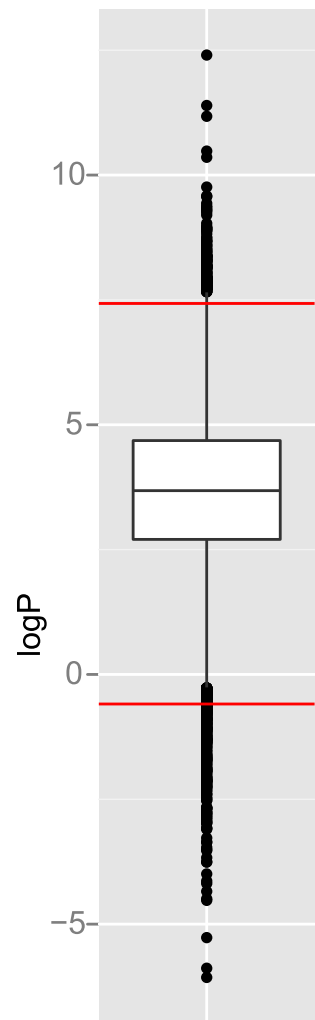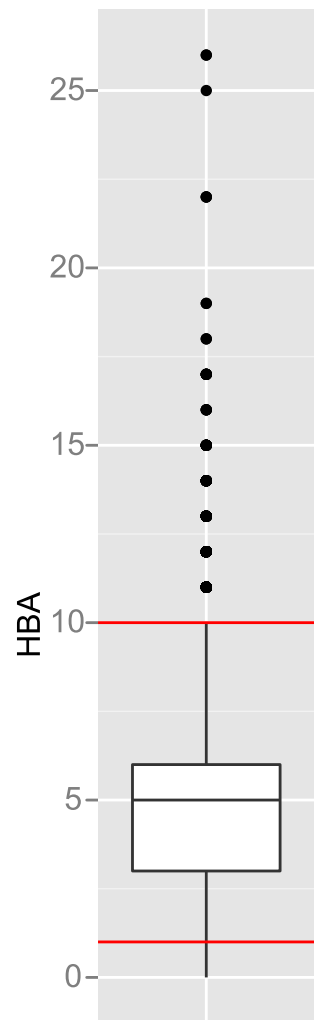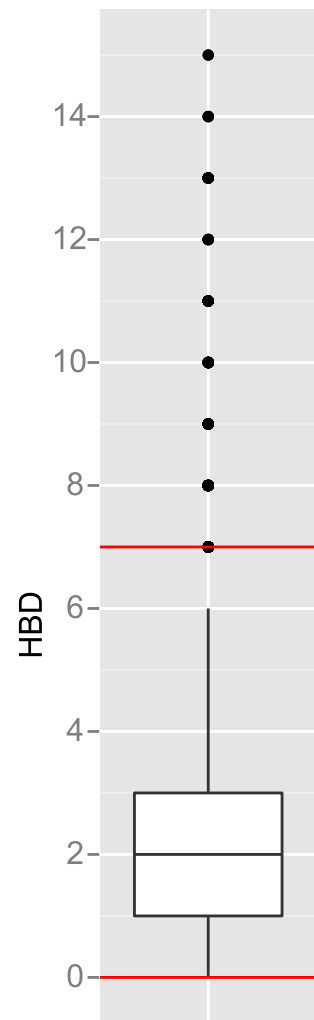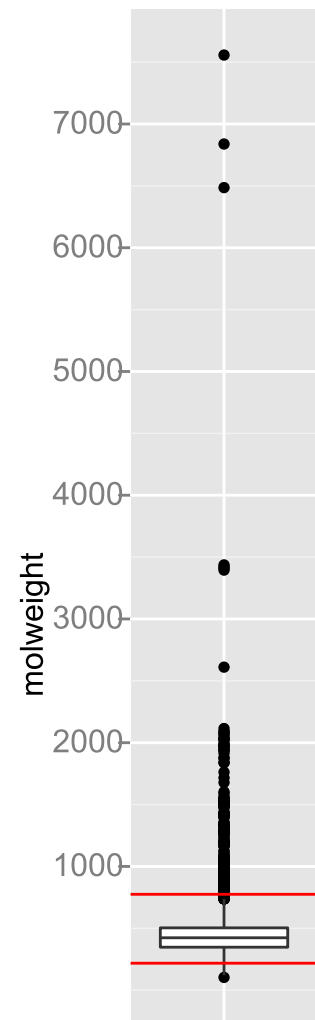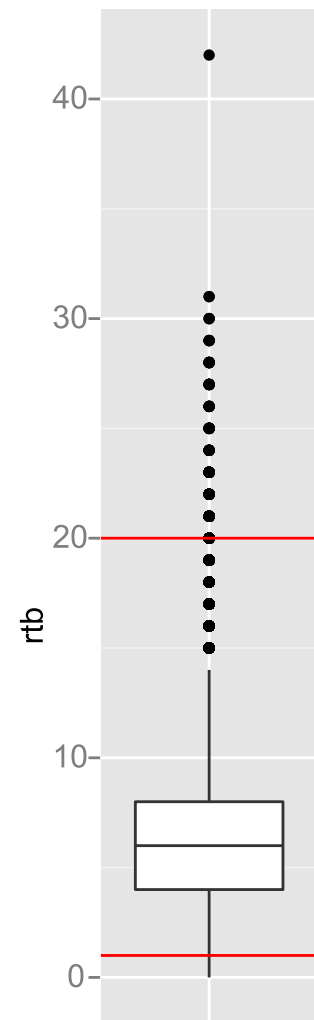

Supplement: Additional file 5 — Outlier selection for PCA. Boxplots show distributions of descriptor values for all molecules in the analysis. Red lines indicate chosen cut-offs for outlier selection. [file 1471-2105-13-S17-S11-S5.pdf]
