# Supplementary material for: Mapping small molecule binding data to structural domains
Source: BMC Bioinformatics. 2012 Dec 7;13(Suppl 17):S11. doi: 10.1186/1471-2105-13-S17-S11 (PMC3521243; doi:10.1186/1471-2105-13-S17-S11)
Supplement: Additional file 7 — Manually removed protein targets. This is a text file listing all entries that were manually removed before mapping small molecule binding. Reasons for the removal are indicated for each identifier. [file 1471-2105-13-S17-S11-S7.pdf]

Additional File 7 – A list of protein targets that were removed from the input data for the mapping algorithm.

Q80T35 - This fragment of the mouse metabotropic glutamate receptor 6 contains only a 7tm_3 domain. All of the eight ChEMBL activities mapping to this target are extracted from a single journal article (Pubmed: 9526567). Nothing in this article indicates that only a fragment of the protein was used and we expect that the binding site should be within the ANF_receptor domain, not the 7tm_3 domain.

Q91755 - This fragment of the X.laevis Glutamate receptor, ionotropic kainate 2 contains only a Lig_chan domain. All of the six ChEMBL activities mapping to this target are extracted from a single journal article (Pubmed: 12672235). Nothing in this article indicates that only a fragment of the protein was used in the assays specified and we expect that the binding site should be within the ANF_receptor of Lig_chan_Glu_bd domains, not the Lig_chan domain.

Q864F1 - This fragment of the porcine Phosphodiesterase isozyme 5 contains only a GAF domain. All of the 237 ChEMBL activities mapping to this target are extracted from one of 10 journal articles (Pubmed: 12672235, 8388468, 8709099, 10891111, 8201604, 9719589, 12570368, 8120866, 8027992, 8254606, 15780616). Nothing indicates that only a fragment of the protein was used in the assays specified and we expect that the binding site should be within the PDEase_I domain, not the GAF domain.

A1Z199 - This fragment of the BCR/ABL p210 fusion protein contains only a SH3_1 domain. All of the 49 ChEMBL activities mapping to this target are extracted from one of 3 journal articles (Pubmed: 19610618, 20188579, 16415863). Nothing indicates that only a fragment of the protein was used in the assays specified and we expect that the binding site should be within the Pkinase_Tyr domain, not the SH3_1 domain.

O46399 - This fragment of Tubulin contains only a Tubulin_C domain. All of the 14 ChEMBL activities mapping to this target are extracted from a single journal article (Pubmed: 9804687). Nothing indicates that only a fragment of the protein was used in the assays specified and we expect that the binding site should be within the Tubulin domain, not the Tubulin_C domain.
